# Microstructure Evolution of the Interface in SiC_f_/TiC-Ti_3_SiC_2_ Composite under Sequential Xe-He-H Ion Irradiation and Annealing Process

**DOI:** 10.3390/nano14201629

**Published:** 2024-10-11

**Authors:** Penghui Lei, Qing Chang, Mingkun Xiao, Chao Ye, Pan Qi, Fangjie Shi, Yuhua Hang, Qianwu Li, Qing Peng

**Affiliations:** 1 School of Nuclear Science and Technology, Xi’an Jiaotong University, Xi’an 710049, China; penghuilei@xjtu.edu.cn; 2 Institute of Clean Energy, Yangtze River Delta Research Institute, Northwestern Polytechnical University, Taicang 215400, China; changqing@mail.nwpu.edu.cn (Q.C.); xiaomingkun@mail.nwpu.edu.cn (M.X.); 3 School of Materials Science and Engineering, Northwestern Polytechnical University, Xi’an 710072, China; 4 China Nuclear Power Operation Technology Corporation, Ltd., Wuhan 430223, China; qipan@cnnp.com.cn; 5 Suzhou Nuclear Power Research Institute, Suzhou 215004, China; fangjie158@163.com (F.S.); hangyuhua@163.com (Y.H.); l1192177798@163.com (Q.L.); 6 State Key Laboratory of Nonlinear Mechanics, Institute of Mechanics, Chinese Academy of Sciences, Beijing 100190, China; 7 Center of Materials Science and Optoelectronics Engineering, University of Chinese Academy of Sciences, Beijing 100049, China; 8 Guangdong Aerospace Research Academy, Guangzhou 511458, China

**Keywords:** SiC_f_/TiC interface, ion irradiation, phase transformation, interface cracking, bubble

## Abstract

A new type of SiC_f_/TiC-Ti_3_SiC_2_ composite was prepared by the Spark Plasma Sintering (SPS) method in this work. The phase transformation and interface cracking of this composite under ion irradiation (single Xe, Xe + He, and Xe + He + H ions) and subsequent annealing were analyzed using transmission electron microscopy (TEM), mainly focusing on the interface regions. Xe ion irradiation resulted in the formation of high-density stacking faults in the TiC coatings and the complete amorphization of SiC fibers. The implanted H ions exacerbated interface coarsening. After annealing at 900 °C for 2 h, the interface in the Xe + He + H ion-irradiated samples was seriously damaged, resulting in the formation of large bubbles and cracks. This damage occurred because the H atoms reduced the surface free energy, thereby promoting the nucleation and growth of bubbles. Due to the absorption effect of the SiC_f_/TiC interface on defects, the SiC fiber areas near the interface recovered back to the initial nano-polycrystalline structure after annealing.

## 1. Introduction

The accident at the Fukushima nuclear power plant revealed that metallic materials have many potential hazards under accidental working conditions, such as metal–water reactions, hydrogen decrepitation, corrosion, etc. [1,2]. In recent decades, some accident-tolerant fuel (ATF) cladding materials have been developed, such as Cr-coated zirconium alloys and FeCrAl, but the neutron economy of Cr is poor [3]. The Cr element can diffuse along grain boundaries under high-temperature steam environments, leading to cladding failure [4]. On the other hand, fiber-reinforced silicon carbide ceramic composites are also considered some of the most promising new cladding materials capable of replacing traditional alloys [5]. Compared to metallic materials, ceramic matrix composites have better hydroxide corrosion resistance and avoid hydrogen explosions, as they do not produce hydrogen gas when reacting with water under accident conditions. In addition, ceramic matrix composites are convenient to design and optimize by changing the compositions and structures of the interphase, matrix, and reinforcement materials. Boron nitride (BN) [6] and pyrolytic carbon (PyC) [7] are commonly used as interphases to enhance the toughness of SiC_f_/SiC composites; however, the behaviors of these two interphase materials are unsatisfactory under irradiation conditions. Under high-temperature neutron irradiation, the volume swelling of PyC is more serious (swelling rate > 10%) [8], which can result in the formation of interface cracking [9]. On the other hand, BN is considered inadequate for nuclear applications due to its high neutron-absorbing cross-section [10]. Thus, it is necessary to find a new interfacial phase material with better irradiation tolerance.

In recent years, several novel interphases have been designed. For example, Agarwal et al. [11] reported that TiC maintains a pristine crystal structure under neutron irradiation at doses of up to 2 dpa at temperatures of 220 °C, 620 °C, and 1150 °C. Therefore, the use of TiC as an interfacial phase material may enhance the irradiation resistance of SiC_f_ composites. Inspired by the properties of TiC, titanium carbide coating was successfully in situ synthesized on silicon carbide fibers to serve as a novel interfacial phase in our previous work [12]. In addition, due to their unique layered structure and excellent irradiation tolerance, a novel class of ternary layered nitrides or carbides, known as MAX phase materials, has also been considered as a potential material for nuclear reactors [13,14,15]. As a typical MAX phase, Ti_3_SiC_2_ has good fracture toughness and good chemical compatibility with silicon carbide [16]. Thus, Ti_3_SiC_2_ could also be designed as a new interfacial material for SiC_f_ composites. Many prior studies [17,18] have reported various methods for preparing SiC_f_ composites with Ti_3_SiC_2_ as an interphase or matrix material. Unfortunately, the irradiation effects of these novel interface structures have not been studied in detail.

In this study, SiC_f_/TiC-Ti_3_SiC_2_ composites were prepared by SPS to evaluate the irradiation resistance of the SiC_f_/TiC-Ti_3_SiC_2_ interface structure. SPS, as a field-assisted sintering technology (FAST), has been applied for fabricating nuclear materials [19,20,21,22,23,24,25]. Ion irradiation, an economical and efficient method, was used to introduce irradiation damage into the SiC_f_/TiC-Ti_3_SiC_2_ interface. The microstructure evolutions of the SiC_f_/TiC-Ti_3_SiC_2_ interface structure under three different irradiation conditions (Xe, Xe + He, Xe + He + H) and the subsequent 900 °C annealing process were analyzed and have been discussed at length.

## 2. Materials and Methods

### 2.1. Materials

The reinforcement used in this study was the 3rd polymer-derived SiC fibers with 0.45 wt% oxygen content. Silicon powder (99.8 wt%) and titanium powder (99.8 wt%) were purchased from Shanghai Aladdin Biochemical Technology Co., Ltd., Shanghai, China; sodium chloride, graphite, and potassium chloride were purchased from Shanghai Macklin Biochemical Technology Co., Ltd., Shanghai, China. The materials are summarized in Table 1. Ti_3_SiC_2_ powders were synthesized using the molten salt synthesis method, and SiC_f_/TiC-Ti_3_SiC_2_ composites were prepared using the SPS method. Compared to conventional unpressurized sintering reactions, molten salts can accelerate atom diffusion to enhance the ceramic sintering reaction, which could accelerate the nucleation of products and reduce the reaction temperature. Simultaneously, large amounts of molten salts can form a liquid environment to control the composition of the products [17,26,27,28].

### 2.2. Preparation of SiC_f_/TiC-Ti_3_SiC_2_ Interface

Ti, Si, and C powders were first pre-mixed in a molar ratio of 3:1:2. Subsequently, each mole of the Ti-Si-C pre-mixed powder was combined with NaCl-KCl in a 1:1 molar ratio. The final powder mixtures were mixed in a planetary ball mill with zirconia grinding balls and ethanol for 24 h. After drying and sieving, the powder was placed in an alumina crucible, heated to 1300 °C in a high-vacuum furnace at a ramp rate of 5 °C/min, and then held at 1300 °C for 3 h. Ti_3_SiC_2_ powders were obtained after washing and drying. Following this, a 10% volume fraction of short-cut silicon carbide fibers and the obtained Ti_3_SiC_2_ powders were mixed and placed into a graphite die with an inner diameter of 10 mm; then, the sintering was conducted using an SPS facility at 1300 °C under a pressure of 40 MPa for 10 min. SiC_f_/TiC-Ti_3_SiC_2_ composites were obtained after cooling the sinter chamber to room temperature.

### 2.3. Ion Irradiation

**Figure 1:**
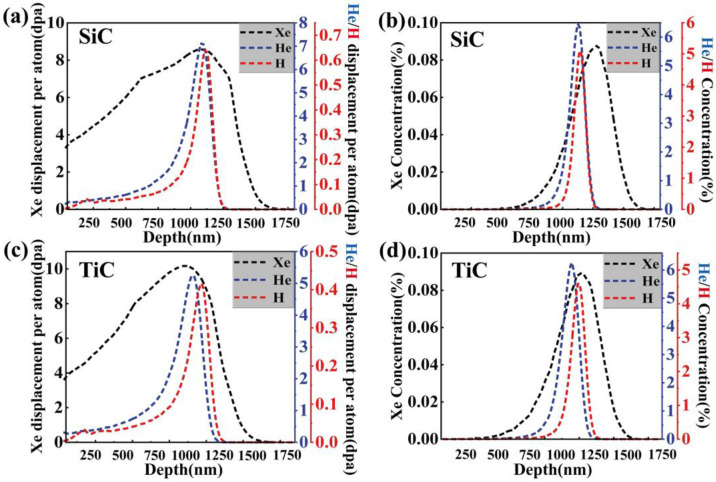
The results of dpa and concentrations (H, Xe, and He) in SiC and TiC calculated by SRIM 2013.

Firstly, 5 MeV Xe^23+^ ions were pre-implanted into the samples at room temperature with a fluence of 3.5 × 10^15^ ions/cm^2^ using a 320 kV ion implanter at the Institute of Modern Physics, Chinese Academy of Sciences. Subsequently, the 400 keV He^+^ and 200 keV H^+^ ion irradiation studies, with fluences of 1 × 10^17^ ions/cm^2^ and 6 × 10^16^ ions/cm^2^, respectively, were conducted at room temperature using an NEC 400 kV ion implanter at Xiamen University. The energies of the ion beams were carefully selected to ensure that the peak concentrations and displacement damage of Xe, He, and H were located in adjacent regions. The ion incidence direction was perpendicular to the sample’s surface. The displacement threshold energies for the elements Ti, Si, and C were set to 25 eV, 15 eV, and 28 eV, respectively, in the Stopping and Range of Ions in Matter (SRIM) 2013 software, based on full damage cascades mode [29]. The density of SiC and TiC was set to 3.23 g/cm^3^ and 4.92 g/cm^3^ in the SRIM calculations, respectively. According to Figure 1, which presents the comprehensive simulation results for the concentrations of He, Xe, and H atoms in SiC and TiC and the depth of damage, the peak hydrogen concentration, peak helium concentration, and peak displacement damage are all located at a depth of around 1 µm. Several irradiation samples were protected with argon gas (purity: 99.999%) and annealed at 900 °C for two hours in a tube furnace. Table 2 outlines the specific experimental conditions employed in this study.

### 2.4. Characterization Methods

Scanning transmission electron microscopy (STEM) with the Talos F200X and scanning electron microscopy (SEM) with the Zeiss Gemini 460 were used to characterize the surface morphologies and microstructures of these unirradiated samples. The phase constitution was analyzed by X-ray diffraction (XRD) using the Bruker D8 ADVANCE at a scan rate of 10°/min. The cross-sectional TEM samples, with a thickness of less than 100 nm, were prepared using a focused ion beam (FIB) with the FEI Helios G4, employing the lift-out technique. In the initial lift-outs, 30 kV Ga^+^ ions with a 9.3 nA current were used. To minimize surface damage to the samples throughout the thinning process, the current of the Ga^+^ ion beam and the energy were gradually reduced, with 2 kV Ga^+^ ions used for the final thinning. The damaged regions are indicated in the overview image of the TEM sample prepared by FIB.

## 3. Results

### 3.1. Characterization of SiC_f_/TiC-Ti_3_SiC_2_ Composites

**Figure 2:**
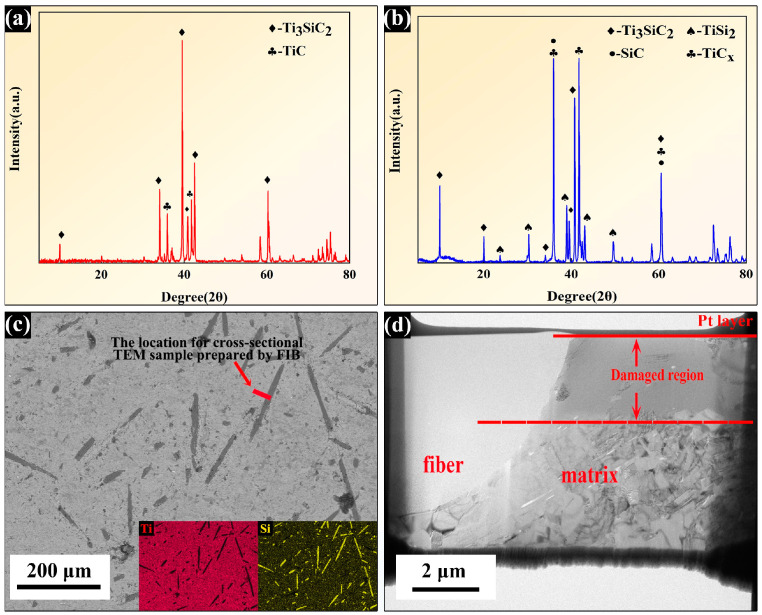
The XRD patterns of the synthesized Ti_3_SiC_2_ powders (**a**) and the SiC_f_/TiC-Ti_3_SiC_2_ composites (**b**); (**c**) the SEM image of the surface morphologies of the as-received composites and the corresponding EDS mappings; (**d**) the overview TEM image of the TEM sample prepared by FIB.

The XRD pattern of the as-prepared Ti_3_SiC_2_ powders is observed in Figure 2a which indicates that the primary phase in the powders is Ti_3_SiC_2_, with small amounts of TiC impurities. The phase compositions of the as-received SiC_f_/TiC/Ti_3_SiC_2_ composites synthesized by SPS are shown in Figure 2b. The peaks at 10°, 20°, 39.6°, and 40.8° correspond to the (002), (004), (104), and (008) crystal planes of Ti_3_SiC_2_. Some peaks at 35.9°, 41.7°, and 60.5° correspond to the (111), (020), and (022) crystal planes of TiC, respectively. Diffraction peaks of the SiC fibers are located at 30° and 60°, overlapping with the diffraction peaks of TiC. Small amounts of TiSi_2_ were also detected. After sintering, the diffraction peaks of TiC were sharp and intense, which indicates that a highly crystalline TiC phase was generated during the sintering process. The polished surface of the as-received composites is shown in Figure 2c, revealing that the SiC fibers are uniformly distributed throughout the Ti_3_SiC_2_ matrix, which exhibits good densification. In addition, the Ti_3_SiC_2_ matrix and the SiC fibers are well bonded. The inserted images in Figure 2c show the corresponding EDS mapping results for this region; the matrix and fiber regions can be clearly distinguished via the distribution of the Ti and Si elements. The irradiated and unirradiated areas, as well as the fiber and matrix regions, can also be easily distinguished by the differences in contrast in Figure 2d.

**Figure 3:**
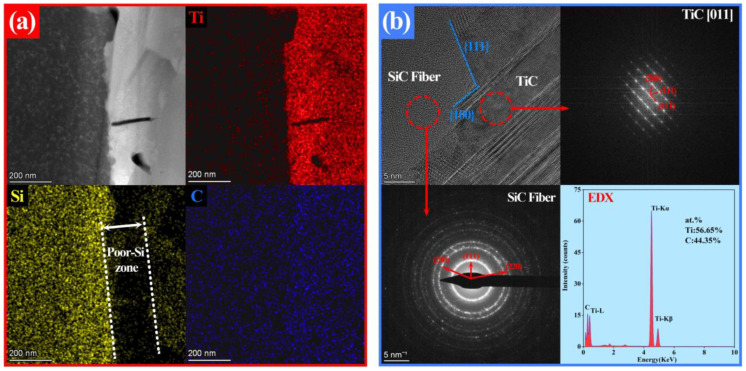
(**a**) The HAADF image and EDX mappings of the SiC_f_/TiC interface; (**b**) the HRTEM image and FFT patterns of the SiC_f_/TiC interface, with the composition near the SiC fibers shown in the EDX spectrum.

Figure 3a presents a high-angle annular dark field (HAADF) image of the interface region along with its EDX mapping results. A poor-Si zone with a thickness of about 150 nm can be seen close to the SiC fiber. The diffraction rings in Figure 3b indicate that the fibers consist of nano-SiC grains. The SAED pattern and the EDX spectrum in Figure 3b illustrate that the composition of this poor-Si zone is the TiC phase with an FCC structure. The HRTEM image in Figure 3b displays a terraced interface structure, with the TiC region near the interface containing numerous stacking faults and nano-twins.

### 3.2. Microstructure Evolutions of the Irradiated SiC_f_/TiC Interface before and after Annealing

**Figure 4:**
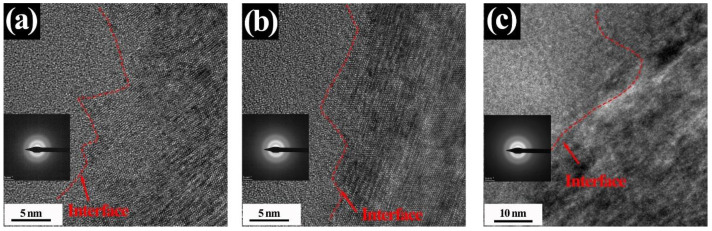
HRTEM images of the SiC_f_/TiC interface regions. Xe irradiation (**a**); Xe + He irradiation (**b**); Xe + He + H irradiation (**c**). Each image includes the SAED patterns of the SiC fiber.

**Figure 5:**
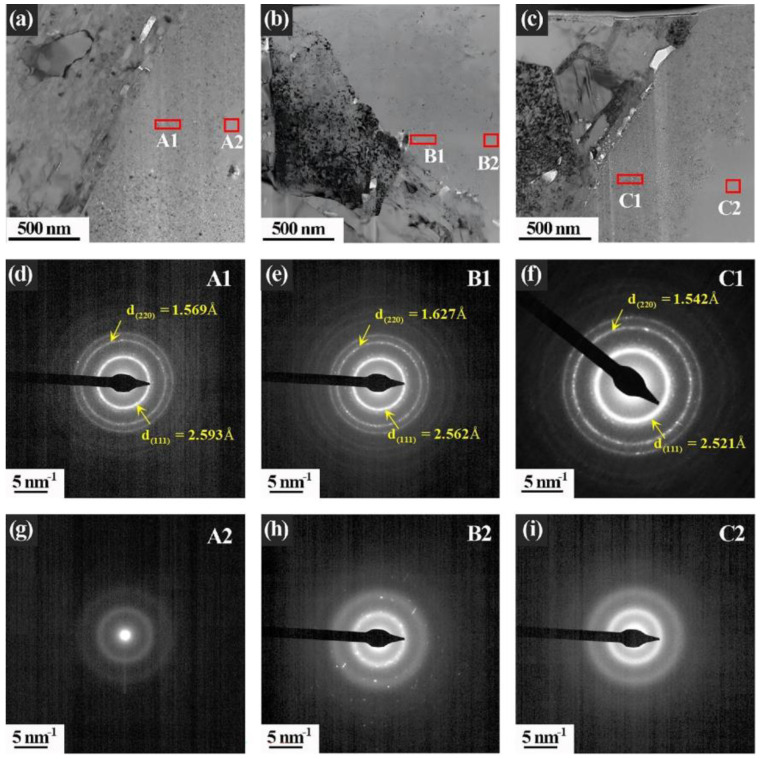
TEM images of irradiated samples post-annealing process: (**a**) irradiation with Xe; (**b**) irradiation with Xe + He; (**c**) irradiation with Xe + He + H; (**d**–**i**) the SAED patterns of A1, B1, C1, A2, B2, and C2 areas marked in (**a**–**c**), respectively.

The HRTEM images of the SiC_f_/TiC interface areas with peak damage under three different irradiation conditions (Xe + He + H, Xe + He, and Xe) are shown in Figure 4a–c. The SAED patterns of the SiC fibers in these three ion-irradiated samples reveal that the SiC fibers are completely amorphized. Under the irradiation of Xe + He and Xe ions, the terraced interface morphologies are still clearly distinguishable. The density of nano-twinning structures and stacking faults in TiC is significantly higher than that in the unirradiated samples. Conversely, under the Xe + He + H irradiation condition, the terraced structure of the SiC_f_/TiC interface is hardly recognizable, which indicates that the implanted H ions could destroy the terraced structure. Following a two-hour annealing at 900 °C, the microstructure of the SiC_f_/TiC interface regions under three irradiation conditions is shown in Figure 5a–c. In the samples irradiated with single Xe ions, the interface remains intact, with no cracks observed, indicating that the injection of single Xe ions has minimal impact on the integrity of the interface after the annealing process. However, small bubbles and cracks were observed in the interface area of the samples injected with H and He ions. Particularly, with the introduction of H ions, a series of cavities were formed at the interface, which indicates that the implanted H ions resulted in more serious damage during the annealing process. Figure 5d–f show the SAED patterns of areas A1, A2, and A3 marked in Figure 5a–c, which indicate that SiC fibers near the interface have recrystallized. Meanwhile, the central areas of the SiC fibers, which are away from the interfaces in these three irradiated composites, are still in an amorphous state after annealing, as shown in Figure 5g–i.

## 4. Discussion

### 4.1. The Decomposition of Ti_3_SiC_2_ and the Formation of TiC Coatings

The decomposition of Ti_3_SiC_2_ can be explained by two important factors. Firstly, the high-temperature and high-pressure environment rendered Ti_3_SiC_2_ unstable, leading to the decomposition of Ti_3_SiC_2_. Compared to Ti–C bonds, the Si–Ti bonds are easier to break. As a result, Si atoms have a tendency to diffuse from the pristine lattice, causing the Ti_3_SiC_2_ phases to decompose into TiC during the sintering process [30,31]. Secondly, graphite molds are capable of reacting with Ti_3_SiC_2_ at high temperatures and pressures, and the infiltration of carbon can intensify the decomposition process, as shown in the following reaction: Ti_3_SiC_2_ + (3X − 2)C → TiC_X_ + Si(g) [32]. The SiC_f_/TiC interface region was formed by several reactions: the high-temperature/-pressure environment and SiC fibers promoted the decomposition of Ti_3_SiC_2_ into TiC(s), Si(g), and Ti(g); then, Ti reacted with the SiC fibers to form TiC films. Meanwhile, partial Si atoms diffused into the matrix and reacted with free Ti atoms to form TiSi_2_. These processes can be expressed as the following reactions: Ti_3_SiC_2_ (s) → TiC_X_(s) + Ti(g) + Si(g); SiC_f_(s) + Ti(g) → TiC_X_(s) + Si(g) [33].

### 4.2. The Microstructure Evolution of Irradiated SiC_f_/TiC Interface Structure

In an irradiation environment, the irradiation damage could interplay with the interfacial interactions to control the grain boundary motions [34], which is also the reason for the structure evolution of the heterogeneous SiC_f_/TiC interfaces. Firstly, during the thermal phase of the cascade evolution process, the incident ions caused atomic rearrangement and localized melting as rapid ion–atom collisions heated and melted the local lattice. Some atoms may have been displaced outside the thermal areas of the cascades and turned into interstitial defects following core solidification, even though most atoms in the cascade’s core were resolidified into the crystal lattice. Then, those interstitial defects accumulated at the boundaries and changed the morphology of localized regions and the roughness of grain boundaries. The roughness became more pronounced after multiple ion impact events. Due to the extremely high migration rate of hydrogen atoms within the lattices, the implanted hydrogen ions, in addition to introducing a small amount of irradiation damage, also accelerated the aggregation of interstitial atomic defects in the interface region, which in turn exacerbated the interface coarsening.

### 4.3. The Phase Transformation and Cracking of Irradiated SiC_f_/TiC Interface Structure after 900 °C Annealing

Zinkle et al. [35] illustrated that temperature plays a crucial role in the microstructural evolution of irradiated samples. At high temperatures, irradiation-induced vacancies and point defects are compounded and annihilated, resulting in a large reduction in the number of defects within the sample and a return of the amorphous structure to its original crystalline structure. The phase interface can act as a strong absorption sink for irradiation-induced defects. Thus, during the annealing process, numerous defects induced by irradiation, such as vacancy clusters, hydrogen atoms, and helium atoms, are prone to accumulate in the interface region. As a result, the SiC fiber regions near the interface returned to their original nano-polycrystalline structure, while other regions of the SiC fiber remained amorphous after annealing since the defects did not have enough time to migrate to the interface region. Hobbs et al. [36] reported that the amorphization of ceramics with covalent bonding is a common response to high-density defect perturbation induced by irradiation and is strongly related to the structural topology. At last, when the total number of defects reached a certain threshold, cracking formed at the interface region.

**Figure 6:**
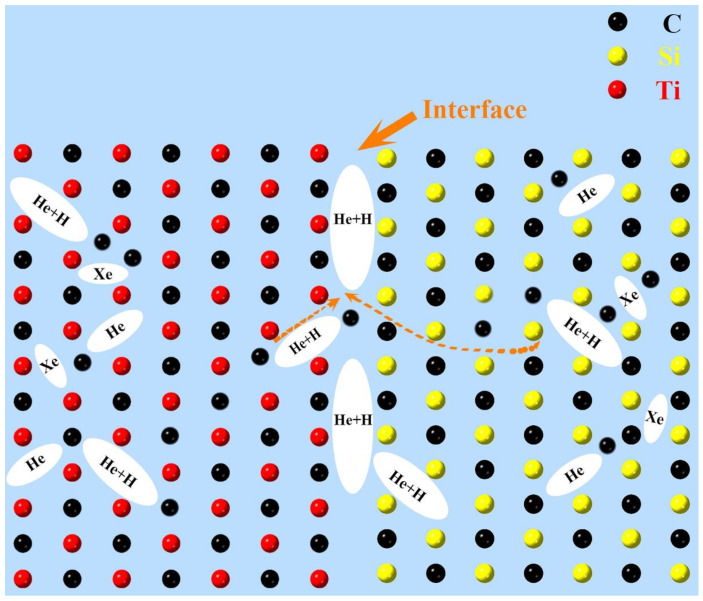
Schematic of bubble aggregation and migration in the interface region of the SiC_f_/TiC sample irradiated with Xe + He + H ions after annealing.

On the other hand, as Xe atoms have a large atomic radius and a high migration energy barrier, most of them were pinned in the SiC/TiC grains and grain boundaries. Therefore, no cavities or Xe bubbles were observed at the interface region in the single Xe ion-irradiated sample. With subsequent high-dose helium ion irradiation, helium bubbles prefer to nucleate at the interface region to form large bubbles [37]. Compared with the bubbles in the Xe + He ion-irradiated SiC fibers, there is a significant increase in the bubble density after the implantation of H ions, as illustrated in Figure 5b,c. The diffusion coefficient dependence on temperature is generally expressed as follows [38]: $D=D_{0}\exp\;(-\;\frac{Q_{A}}{\mathrm{kT}})$, where *D_0_* is the constant; *Q_A_* represents the activation energy for diffusion, which is positively correlated with atomic size; and *k* and *T* are the Boltzmann constant and temperature, respectively. Therefore, the increased bubble density following the implantation of H ions is related to the higher diffusion coefficient of H compared to those of He and Xe. Our previous study [39] illustrates that the implanted H ions have a fast migration rate inside the lattice and can attach to He bubbles to accelerate the migration of He bubbles to the interface; in addition, helium bubbles nucleate and grow more readily because H ions can reduce their surface energy [40]. The nucleation of bubbles is affected by the combination of volume free energy and surface energy, which can be described by the following equation [41]: $∆G=\frac{4}{3}\pi r^{3}∆G_{v}+4\pi r^{2}\gamma$, where ∆G is the total free energy change; $∆G_{v}$ represents the free energy per unit volume, which becomes negative during nucleation; *r* represents the radius of an embryo, and when the embryo radius is larger than the critical nucleus radius r^*^, nucleation is triggered; and *γ* represents the specific surface energy, serving as a retardation force with a surface energy of $4\pi r^{2}\gamma$. Due to the reduction in surface energy induced by implanted H ions, the critical nucleus radius r^*^ of the bubbles decreased in the sample irradiated with Xe + He + H ions, which means that the nucleation and growth of helium bubbles become easier. Thus, the average size of bubbles in the interface region under Xe + He + H ion irradiation is larger than that in samples irradiated with only Xe + He or Xe ions. Figure 6 presents a schematic of bubble aggregation and migration in the interface region of the sample irradiated with Xe + He + H ions. Hydrogen bubbles are formed by the reaction between incident H ions and electrons from the sample [42,43], as represented by the following chemical reaction formula: H_2_ = 2H^+^ + 2e^−^.

The results of this work indicate that He and H atoms have a more serious impact on the material interface, and the annealing process is helpful for the recrystallization recovery of the irradiation-induced amorphous interface regions. Overall, this work suggests that a material’s resistance to radiation can be enhanced through rational interface design. Our future work will focus on improving the radiation swelling and cracking resistance of the interface in SiC fiber composite materials.

## 5. Conclusions

SiC_f_/TiC-Ti_3_SiC_2_ composites prepared using the SPS method were irradiated with single Xe, Xe + He, and Xe + He + H ions. Then, the microstructure evolutions of the SiC_f_/TiC interface before and after annealing were investigated. The following summarizes the primary conclusions:

(1) The behaviors of the SiC_f_/TiC interface under three different ion irradiation conditions (single Xe, Xe + He, and Xe + He + H) were researched. After irradiation, the SiC fibers became completely amorphous, while partial amorphization and high-density stacking faults were observed in TiC. The implanted H ions introduced more damage, thereby exacerbating the interface coarsening.
(2) After 900 °C annealing, the implanted He and H ions preferred to accumulate in the SiC_f_/TiC interface region and formed bubbles. The H ions reduced surface free energy, thereby promoting the nucleation and growth of bubbles, which caused cracking. Due to the strong absorption effect of the interface on irradiation defects, the SiC fiber regions near the interface returned to their original nano-polycrystalline structure, which suggests that the materials’ resistance to radiation can be enhanced through rational interface designs.

## Figures and Tables

**Table 1:** Chemicals used in this study.

Materials	Purity	Supplier
Ti	99.8%	Aladdin lnc., Shanghai, China
Si	99.9%	Aladdin lnc., Shanghai, China
graphite	99.95%	Macklin Inc., Shanghai, China
NaCl	99.8%	Macklin Inc., Shanghai, China
KCl	99.9%	Macklin Inc., Shanghai, China
ethanol	99.70%	Sinopharm Chemical Reagent Co., Ltd., Shanghai, China

**Table 2:** The detailed ion irradiation experimental conditions used in this work.

Influence (Ions/cm^2^)			Temperature for Annealing (°C)	Time for Annealing (h)
Xe Ions	He Ions	H Ions		
3.5 × 10^15^			900	2
3.5 × 10^15^	1 × 10^17^			
3.5 × 10^15^	1 × 10^17^	6 × 10^16^		

## Data Availability

The data presented in this study are available on request from the corresponding authors.

## References

[1] Ni N., Hudson D., Wei J., Wang P., Lozano-Perez S., Smith G., Sykes J., Yardley S., Moore K., Lyon S. (2012). How the crystallography and nanoscale chemistry of the metal/oxide interface develops during the aqueous oxidation of zirconium cladding alloys. Acta Mater..

[2] Mazères B., Desgranges C., Toffolon-Masclet C., Monceau D. (2016). Experimental study and numerical simulation of high temperature (1100–1250 °C) oxidation of prior-oxidized zirconium alloy. Corros. Sci..

[3] Kam D.H., Lee J.H., Lee T., Jeong Y.H. (2015). Critical heat flux for SiC-and Cr-coated plates under atmospheric condition. Ann. Nucl. Energy.

[4] Yamamoto K., Takayama T., Minamino Y., Koizumi Y., Tokunaga T., Hagihara K. (2023). Modification of grain boundary microstructure by controlling dissolution behavior of θ particles in Cr-containing hypereutectoid steel. Mater. Charact..

[5] Zinkle S.J., Terrani K.A., Gehin J.C., Ott L.J., Snead L.L. (2014). Accident tolerant fuels for LWRs: A perspective. J. Nucl. Mater..

[6] Shan Q., Xu Q., Xue Y., Lian J., Wang Y., Chen C., Zhou Y., Ma Q., Shui A. (2021). The tensile damage behavior of SiC_f_/SiC–B4C after oxidation in wet atmosphere based on acoustic emission pattern recognition. J. Am. Ceram. Soc..

[7] Fellah C., Braun J., Sauder C., Sirotti F., Berger M.-H. (2020). Influence of the carbon interface on the mechanical behavior of SiC/SiC composites. Compos. Part A Appl. Sci. Manuf..

[8] Snead L., Burchell T., Katoh Y. (2008). Swelling of nuclear graphite and high quality carbon fiber composite under very high irradiation temperature. J. Nucl. Mater..

[9] Zhao Y., Li X., Liu C., Yang H., Chen B., Qin Y., Xu S., Cheng L., Zhang L. (2019). Irradiation effects on Amosic-3 silicon carbide composites by Si ions implantation. J. Eur. Ceram. Soc..

[10] Katoh Y., Ozawa K., Shih C., Nozawa T., Shinavski R.J., Hasegawa A., Snead L.L. (2014). Continuous SiC fiber, CVI SiC matrix composites for nuclear applications: Properties and irradiation effects. J. Nucl. Mater..

[11] Agarwal S., Koyanagi T., Bhattacharya A., Wang L., Katoh Y., Hu X., Pagan M., Zinkle S.J. (2020). Neutron irradiation-induced microstructure damage in ultra-high temperature ceramic TiC. Acta Mater..

[12] Yang J., Ye F., Cheng L. (2023). In-situ synthesized nano-porous titanium carbide coating on silicon carbide fibres using titanium tetrachloride vapour. Ceram. Int..

[13] Barsoum M.W. (2000). The M_N+1_AX_N_ phases: A new class of solids: Thermodynamically stable nanolaminates. Prog. Solid State Chem..

[14] Whittle K.R., Blackford M., Aughterson R., Moricca S., Lumpkin G.R., Riley D., Zaluzec N. (2010). Radiation tolerance of M_n+1_AX_n_ phases, Ti_3_AlC_2_ and Ti_3_SiC_2_. Acta Mater..

[15] Liu S., Yang T., Zhang J., Yan Z., Lu Y., Han D., Wang C., Fang Y., Wang Y. (2018). Thermal effects in ion irradiated Ti_2_AlC and Ti_3_SiC_2_. Nucl. Instrum. Methods Phys. Res. Sect. B Beam Interact. Mater. At..

[16] Zhang J.W., Hu C.F., Wang Y.G., Huang Q., Cui P. (2013). Interfacial reactions between polymer derived SiC fiber and Ti_3_Si(Al)C_2_. Key Eng. Mater..

[17] Yang J., Ye F., Cheng L. (2022). In-situ formation of Ti_3_SiC_2_ interphase in SiC_f_/SiC composites by molten salt synthesis. J. Eur. Ceram. Soc..

[18] Tao P., Liu W., Wang Y. (2020). Fabrication of SiC_f_/Ti_3_SiC_2_ composites with high thermal conductivity by spark plasma sintering. Ceram. Int..

[19] Cappia F., Cullison M., Chen T., Kombaiah B., Bawane K., Teng F., Madden J., Perez E., Yao T., Lei P. (2022). Grain subdivision and structural modifications by high-energy heavy ions in UO_2_ with different initial grain size. Nucl. Instrum. Methods Phys. Res. Sect. B Beam Interact. Mater. At..

[20] Yao T., Guo X., Lei P., Wang Y., Frankel G.S., Lian J. (2020). Corrosion interactions between stainless steel and lead vanado-iodoapatite nuclear waste form part II. NPJ Mater. Degrad..

[21] Guo X., Gin S., Lei P., Yao T., Liu H., Schreiber D.K., Ngo D., Viswanathan G., Li T., Kim S.H. (2020). Self-accelerated corrosion of nuclear waste forms at material interfaces. Nat. Mater..

[22] Guo X., Gin S., Lei P., Yao T., Liu H., Schreiber D.K., Ngo D., Viswanathan G., Li T., Kim S.H. (2020). Reply to: How much does corrosion of nuclear waste matrices matter. Nat. Mater..

[23] Lei P., Yang K., Shi T., Wei M., Ran G., Lu C. (2022). Surface alteration and chemical durability of hollandite (Cr, Al and Ti) consolidated by spark plasma sintering in acid solution. J. Nucl. Mater..

[24] Lei P., Yao T., Gong B., Zhu W., Ran G., Lian J. (2020). Spark plasma sintering-densified vanadinite apatite-based chlorine waste forms with high thermal stability and chlorine confinement. J. Nucl. Mater..

[25] Lei P., Ji X., Qiu J., Si J., Peng T., Teng C., Wu L. (2024). The Influence of Grain Size on Microstructure Evolution in CeO_2_ under Xenon Ion Irradiation. Nanomaterials.

[26] Chen J., Ye F., Cheng L., Yang J., Chen X. (2023). Preparation and properties of Ti_3_SiC_2_ preform reinforced SiC ceramic matrix composites. J. Eur. Ceram. Soc..

[27] Kimura T. (2011). Molten salt synthesis of ceramic powders. Advances in Ceramics—Synthesis and Characterization, Processing and Specific Applications.

[28] Dash A., Sohn Y.J., Vaßen R., Guillon O., Gonzalez-Julian J. (2019). Synthesis of Ti_3_SiC_2_ MAX phase powder by a molten salt shielded synthesis (MS3) method in air. J. Eur. Ceram. Soc..

[29] Ziegler J.F., Ziegler M.D., Biersack J.P. (2010). SRIM–The stopping and range of ions in matter (2010). Nucl. Instrum. Methods Phys. Res. Sect. B Beam Interact. Mater. At..

[30] Emmerlich J., Music D., Eklund P., Wilhelmsson O., Jansson U., Schneider J.M., Högberg H., Hultman L. (2007). Thermal stability of Ti_3_SiC_2_ thin films. Acta Mater..

[31] Qin J., He D. (2013). Phase stability of Ti_3_SiC_2_ at high pressure and high temperature. Ceram. Int..

[32] Magnus C., Rainforth W.M. (2019). Spark plasma sintering (SPS) synthesis and tribological behaviour of MAX phase composite of the family Ti_n+1_SiC_n_ (n = 2). Wear.

[33] He G., Xu J., Zhang Z., Qian Y., Zuo J., Li M., Liu C. (2021). Interfacial reactions and mechanical properties of SiC fiber reinforced Ti_3_SiC_2_ and Ti_3_(SiAl)C_2_ composites. Mater. Sci. Eng. A.

[34] Barr C.M., Chen E.Y., Nathaniel J.E., Lu P., Adams D.P., Dingreville R., Boyce B.L., Hattar K., Medlin D.L. (2022). Irradiation-induced grain boundary facet motion: In situ observations and atomic-scale mechanisms. Sci. Adv..

[35] Zinkle S. (2012). 1.03—Radiation-Induced effects on microstructure. Compr. Nucl. Mater..

[36] Hobbs L.W., Clinard F.W., Zinkle S.J., Ewing R.C. (1994). Radiation effects in ceramics. J. Nucl. Mater..

[37] Chen C.-H., Zhang Y., Fu E., Wang Y., Crespillo M.L., Liu C.-Z., Shannon S., Weber W.J. (2014). Irradiation-induced microstructural change in helium-implanted single crystal and nano-engineered SiC. J. Nucl. Mater..

[38] Pramono Y., Sasaki K., Yano T. (2004). Release and diffusion rate of helium in neutron-irradiated SiC. J. Nucl. Sci. Technol..

[39] Ye C., Xue J., Liu T., Shu R., Yan Y., Liao Y., Ren Q., Ran G., Sun K., Jiang L. (2020). The microstructure evolution in a SiC_f_/SiC composite under triple ion beam irradiation. Ceram. Int..

[40] Liu Y.-L., Zhang Y., Zhou H.-B., Lu G.-H., Liu F., Luo G.-N. (2009). Vacancy trapping mechanism for hydrogen bubble formation in metal. Phys. Rev. B Condens. Matter Mater. Phys..

[41] Jastrzebski Z.D., Komanduri R. (1988). The Nature and Properties of Engineering Materials.

[42] Sun J., You Y.-W., Hou J., Li X., Li B., Liu C., Wang Z. (2017). The effect of irradiation-induced point defects on energetics and kinetics of hydrogen in 3C-SiC in a fusion environment. Nucl. Fusion.

[43] Dong W., Shen Q., Wei M., Lei P., Song L., Chang Q., Ye C. (2023). Research on the surface damage of Si^+^ and H^+^ co-implanted 6H-SiC before and after annealing. Nucl. Instrum. Methods Phys. Res. Sect. B Beam Interact. Mater. At..

